# On the noisy spatiotopic encoding of word positions during reading: Evidence from the change-detection task

**DOI:** 10.3758/s13423-020-01819-3

**Published:** 2020-10-09

**Authors:** Felipe Pegado, Jonathan Grainger

**Affiliations:** 1 Laboratoire de Psychologie Cognitive, CNRS and Aix-Marseille University, 3 place Victor Hugo, 13331 Marseille, France; 2 Institute for Language Communication and the Brain, Aix-Marseille University, Marseille, France

**Keywords:** Reading, Word position, Spatiotopic representations, Change detection, Transposed words

## Abstract

The present study builds on our prior work showing evidence for noisy word-position coding in an immediate same-different matching task. In that research, participants found it harder to judge that two successive brief presentations of five-word sequences were different when the difference was caused by transposing two adjacent words compared with different word replacements – a transposition effect. Here we used the change-detection task with a 1-s delay introduced between sequences – a task thought to tap into visual short-term memory. Concurrent articulation was used to limit the contribution of active rehearsal. We used standard response-time (RT) and error-rate analyses plus signal detection theory (SDT) measures of discriminability (d’) and bias (*c*). We compared the transposition effects for ungrammatical word sequences and nonword sequences observed with these different measures. Although there was some evidence for transposition effects with nonwords, the effects were much larger with word sequences. These findings provide further support for the hypothesized noisy assignment of word identities to spatiotopic locations along a line of text during reading.

## Introduction

In recent theoretical work on skilled reading the notion of a spatiotopic representation of word positions has taken a central role (Grainger, 2018; Snell, Meeter, & Grainger, 2017; Snell, van Leipsig, Grainger, & Meeter, 2018). Spatiotopic coordinates provide a reference frame for representing the location of an object in a visual scene *independently* of where the viewer’s eyes are looking. Adapted to the case of reading, the spatiotopic coordinates for written words are defined as representing a word’s location in a line of text that is being read, independently of the position of the reader’s gaze on that line of text. Spatiotopic locations are defined by low-level visual information provided by the spaces between words, and, as readers move their eyes along the line of text, word identities are gradually assigned to these locations. In sum, readers perceive a series of visual “blobs” while they are processing word identities during reading, and they associate different word identities with different blob locations (Reilly & Radach, 2006; Snell et al., 2018). Under the hypothesis of parallel word processing (Snell & Grainger, 2019a), a central ingredient of this theoretical framework is that it is the bottom-up association of word identities to spatiotopic locations that provides the crucial information from which word order can be inferred and subsequently used for syntactic processing.^1^

Encoding the relative positions of visual objects is known to be subject to noise. Clear evidence for this has been provided for arrays of letter, digit, and other simple visual stimuli using the same-different matching task (e.g., Gómez, Ratcliff, & Perea, 2008; Massol, Duñabetia, Carreiras, & Grainger, 2013). In this task, participants simply have to judge, as rapidly and as accurately as possible, if two successive stimulus arrays are the same or different. Each array is typically presented briefly in order to make the task harder and in order to place the emphasis on visual processing (the task is sometimes referred to as the “perceptual matching” task, e.g., Ratcliff, 1981). One key finding obtained with this task (see Gómez et al., 2008; Massol et al., 2013) is that it is harder for participants to judge that two strings of elements are different when the difference is generated by transposing two elements (e.g., PFGHK – PFHGK) compared with substituting two elements with different ones (e.g., PFGHK – PFMDK). This has been taken as evidence that the encoding of positional information is subject to a certain amount of noise, such that evidence for a given item at a given location can also be taken as evidence for that item at neighboring locations.

Building on prior work revealing transposed-word effects in a grammatical decision task (Mirault, Snell, & Grainger, 2018; Snell & Grainger, 2019b), in more recent work we reported a transposed-word effect in the same-different matching task (Pegado & Grainger, 2019, 2020). That is, our participants found it harder to decide that two word sequences were different when that difference was generated by transposing two words (e.g., he wants these green apples/he these wants green apples) compared with replacing two words with different words (e.g., he talks their green apples). Crucially, these transposed-word effects were also found with ungrammatical word sequences (e.g., green wants these he apples/green these wants he apples), and the effects found with ungrammatical word sequences were significantly greater than those found with sequences of nonwords (e.g., ergen twans shete eh lapeps/ergen shete twans eh lapeps). The overall pattern of effects allowed us to conclude that the bottom-up noisy association of word identities with spatiotopic locations is one key mechanism underlying transposed-word effects.

In the present study we seek more direct evidence for the spatiotopic nature of bottom-up word-position encoding. To do so we use a paradigm that has been the paradigm of choice for investigating visual short-term memory (VSTM), and the spatiotopic nature of location encoding in VSTM (Luck, 2008; Luck & Hollingworth, 2008) – the change-detection paradigm. Change detection differs from the immediate same-different matching task in terms of the delay between the reference and the target stimuli, which is typically around 1 s, and thought to be long enough to rule out contributions from iconic memory (Vogel, Woodman, & Luck, 2001). We further limit any contribution of active rehearsal by using concurrent articulation (Baddeley, 1986; see Ktori, Grainger, & Dufau, 2012, for an application of this procedure with letter and digit arrays in the change-detection task). One of the key differences between the change-detection paradigm and paradigms used to study other forms of short-term memory, such as verbal short-term memory (vSTM, not to be confused with VSTM), is the brief simultaneous presentation of the to-be-remembered stimuli compared with the longer sequential presentation used in vSTM tasks. The longer sequential presentation used in vSTM paradigms would enable phonological recoding of visual stimuli, hence leading to the general consensus that phonological representations are involved in the short-term storage of verbal information independently of the modality of presentation (e.g., Baddeley, 1986). On the contrary, we hypothesize that orthographic representations are primarily involved in storing words in VSTM. Evidence in support of this hypothesis has recently been reported by Cauchi, Lété, and Grainger (2020). Using the Eriksen flanker task with horizontally aligned target and flankers, Cauchi et al. found that orthographic overlap across target and flanker stimuli, but not phonological overlap, impacted on target-word identification. In sum, we believe that the combination of the change-detection procedure and concurrent articulation provides an ideal means to investigate the parallel association of orthographic word identities to spatial locations.

Apart from building on our prior work with immediate same-different matching (Pegado & Grainger, 2019, 2020), the present study was motivated by the findings of Mirault and Grainger (2020). In that study, grammatical and ungrammatical five-word sequences were presented in random order for a randomly varying duration (followed by a masking stimulus), and 87% accuracy for detecting grammaticality was already attained with 300-ms stimulus exposures. We explained this performance by the parallel processing of multiple word identities and their maintenance in VSTM during the computation of syntactic information. Given our explanation of transposed-word effects as reflecting the noisy association of word identities to spatiotopic locations in VSTM, we therefore expected to find similar effects in the change-detection task, and we expected these effects to be greater for word sequences than nonword sequences. However, the findings of Mirault and Grainger (2020) could be accommodated by a serial processing model (e.g., E-Z Reader: Reichle, Pollatsek, Fisher, & Rayner, 1998) by assuming that it is not word identities but rather lower-level visual or orthographic information that is held in a short-term store. Given the capacity limits of VSTM (e.g., Cowan, 2001), this would have to involve another form of short-term storage that would enable serial word processing in the absence of visual input. That is, in the Mirault and Grainger (2020) study, participants would have had enough time to store sublexical information concerning several words that would have enabled lexical and sentence-level processing to continue upon presentation of the backward mask. Given the constraints of serial word reading, this storage can only involve sublexical representations (which, contrary to lexical representations, can be processed in parallel), and, therefore, transposition effects should be similar for word and nonword sequences under this account.

Finally, we note that although it might appear obvious that change detection should be easier for word sequences than nonword sequences, due, for example, to chunking mechanisms or redintegration impacting on short-term memory, the key prediction that we are testing is that, according to our parallel word-processing account, it is the nature of the change (transposition vs. replacement) that should differentially impact on the ability to detect these changes in word and nonword sequences. This, we believe, is not a trivial prediction.

## Methods

### Participants

Thirty-one^2^ native French speaker participants (29 females) were recruited at Aix-Marseille University (Marseille, France) to take part in this experiment. All reported normal or corrected-to-normal vision, ranged in age from 19 to 29 years (M = 23.3 years, SD = 2.6), and signed informed-consent forms prior to participation. One participant discontinued the experiment. Participants received either monetary compensation (10 €/h) or course credit. Ethics approval was obtained from the Comité de Protection des Personnes SUD-EST IV (No. 17/051).

### Design and stimuli

We created 40 five-word ungrammatical sequences by scrambling the order of words in correct sentences in French. These 40 word sequences were used to generate an equivalent number of nonword sequences by scrambling the order of letters in each word to generate a nonword. The 40 word and 40 nonword sequences formed the set of sequences that were presented as the first of two sequences on each trial, henceforth called the *reference*. For every reference we generated three types of target sequence (the second sequence on each trial), for a total of 240 trials. The three types of target were: (1) repetition – the same sequence as the reference; (2) transposition – the words/nonwords at positions 2 and 3 or positions 3 and 4 in the reference were flipped; (3) replacement – the words/nonwords at positions 2 and 3 or positions 3 and 4 in the reference were replaced with different words/nonwords. The replacement words had the same length, syntactic function, and word frequency (M transposed = 3.51, M replaced = 3.49; p = 0.40)^3^ as the words they replaced. The replacement nonwords were scrambled versions of the replacement words. The average length of the two critical words was 4.54 letters (range 1–6 letters). The design involved distinct analyses for the “same” response trials and the “different” response trials. The “same” response analysis contrasted word and nonword references (Reference Lexicality factor). The “different” response analysis involved a 2 (Reference Lexicality) × 2 (Type of Change) design. Table 1 provides examples of reference and target sequences used in the “different” response conditions in the Experiment (French), and also in English for expository purposes. For each participant, every reference was repeated three times associated with one of its three target sequences (one same response, two types of different response).

**Table 1:** Examples of the reference and target sequences for the “different” response trials

Word sequences		
Reference	vertes veut ces il pommes	green wants these he apples
Transposed Target	VERTES CES VEUT IL POMMES	GREEN THESE WANTS HE APPLES
Replaced Target	VERTES DIRA MES IL POMMES	GREEN TALKS THEIR HE APPLES
Nonword sequences		
Reference	vreste vute cse li pmomes	eh wtasn heste gnere palpse
Transposed Target	VRESTE CSE VUTE LI PMOMES	EH HESTE WTASN GNERE PALPSE
Replaced Target	VRESTE DRAI MSE LI PMOMES	EH SKEIl HOSET GNERE PALPSE

*Note.* Not shown here is the condition where targets had the same stimuli sequence as the reference but were printed in uppercase (i.e., “same” response trials). The transpositions and replacements operate on the second and third words in these examples and could equally be on the third and fourth words in the experiment. English examples are provided for convenience

### Apparatus

Stimuli were presented using OpenSesame (Version 3.0.7; Mathôt, Schreij, & Theeuwes, 2012) and displayed on a 47.5 × 27-cm LCD screen (1,024 × 768-pixel resolution). Participants were seated about 70 cm from the monitor, such that every four characters (monospaced font in black on a gray background) equaled approximately 1° of visual angle. Responses were recorded via a computer keyboard: “j” key (right index finger) for “same” responses and “f” (left index finger) for “different” responses.

### Procedure

**Fig. 1:**
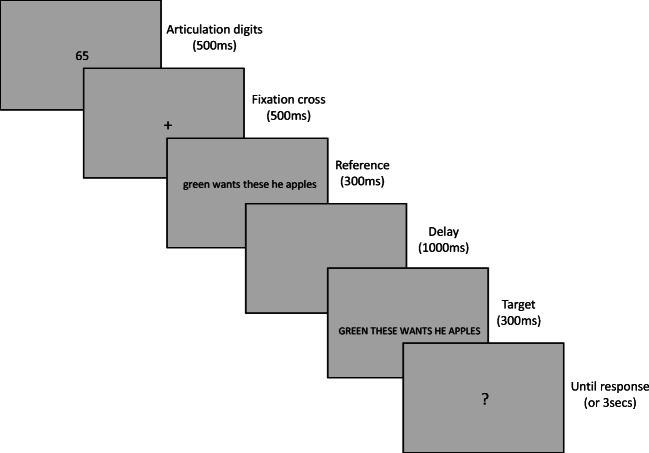
A typical trial. Participants repeatedly read aloud the two digits (e.g., “six,” “five”) presented at the beginning of the trial for the duration of the trial

The experiment took place in a quiet dimly lit room. The instructions were given both by the experimenter and on-screen. On every trial, participants had to decide if the two sequences presented one after the other on the computer screen were the same or different, where “same” was defined as being composed of the same words in the same order. The first sequence, the reference, was always presented in lower case, while the second sequence, the target, was always shown in uppercase, in order to avoid purely visual matching. In order to compensate for the difference in the size of lowercase and uppercase letters, the font size of the reference was slightly greater than that of the target (24 pixels and 22 pixels, respectively), such that one character corresponded to approximately 0.3 cm in both cases. All stimuli were presented in droid monospaced font, the default font for OpenSesame. In the main experiment, each trial started with a fixation cross for 500 ms followed by the reference sequence for 300 ms, followed by a delay without stimuli for 1,000 ms, followed by the target sequence for 300 ms, followed by a question mark “?” presented until the participants responded (or for a maximum of 3 s). Then a neutral gray screen was displayed for 200 ms and a new trial started. Participants were requested to respond as fast and as accurately as possible. For the purposes of concurrent articulation, at the beginning of each trial two random digits were presented for 500 ms and participants were instructed to repeatedly read them aloud during the whole duration of the trial. This procedure was used in order to avoid active rehearsal during the delay between reference and target presentation (see Fig. 1). A training phase was performed before the experiment to familiarize participants with the task. This consisted of 12 trials with a different set of stimuli and longer presentation durations for both reference and target sequences (800 ms each) but the same 1-s delay between them as in the main experiment. Feedback (correct vs. incorrect response) was provided to participants after each trial in the training phase.

### Analysis

Given the speeded nature of the task, we first performed RT and error-rate analyses using linear mixed effects (LME) models. We then applied signal detection theory (SDT; Macmillan & Creelman, 2005) to analyze discriminability (d’) and bias (*c*) in making a “different” response. The statistical analysis of log10 transformed RTs and error rates was performed with R software (version 3.5.1), separately for “same” and “different” responses using the LME4 library (Bates, Maechler, Bolker, & Walker, 2015), declaring participants and items as random variables. We first tried fully randomized mixed models, including random slopes in addition to random intercepts (Barr, Levy, Scheepers, & Tily, 2013), but in several analyses (for Error Rates on “different” trials and RTs on “different” trials), the analyses failed to converge (that is, no solution was found within a reasonable number of iterations). For consistency, we adopted random intercept-only models for all the analyses. We report *b*-values, standard errors (SEs), and *t*-values (for RTs) and *z*-values (for errors), with *t*- and *z*-values beyond |1.96| deemed significant (Baayen, 2008). SDT was used to determine the sensitivity (d’) and response bias (*c*) of participants when detecting each type of change in the “different” response condition.

## Results

### Response times (RTs)

**Fig. 2:**
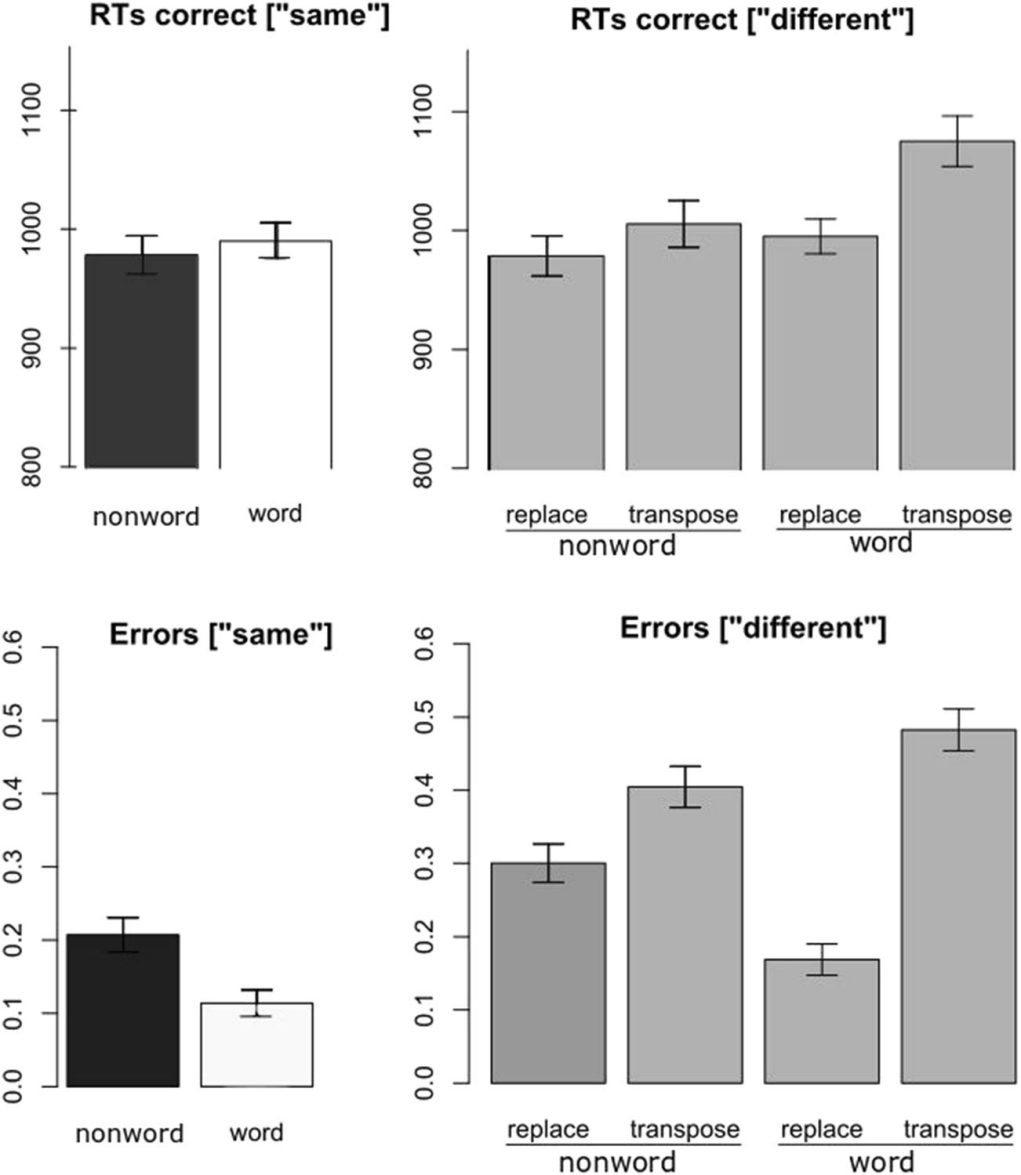
Response times (RTs) in milliseconds (upper panel) and error rates in probabilities (lower panel) for “same” response trials (left) and “different” response trials (right) as a function of reference lexicality (words vs. nonwords) and type of change (replace vs. transpose). Error bars represent 95% confidence intervals

Trials with RTs ± 2.5 SDs were excluded from the analysis prior to log transformation. Condition means are shown in Fig. 2. Participants presented an overall error rate of 28.0% and a mean RT for correct trials of 999 ms.

#### “Same” trials

Reference lexicality did not significantly influence RTs for same trials (*b* = 0.005, SE = 0.004, *t* = 1.15), with the average RT for words being 990 ms and 978 ms for nonwords.

#### “Different” trials

There was a main effect of type of change (*b* = 0.013, SE = 0.005, *t* = 2.87), with slower responses in the transposed condition (1,038 ms) than the replaced condition (987 ms). The main effect of reference lexicality was not significant (*b* = 0.008, SE = 0.004, *t* = 1.84), but there was a significant type of change × reference lexicality interaction (*b* = 0.002, SE = 0.006, *t* = 3.75), with transposition effects being larger for words (33.3 ms: *b* = 0.04, SE = 0.005, *t* = 7.87) than nonwords (12.5 ms: *b* = 0.01, SE = 0.005, *t* = 2.86).

### Error rates

The condition means are shown in Fig. 2.

#### “Same” trials

For trials requiring a “same” response, reference lexicality significantly affected error rates (*b* = 0.74, SE = 0.12, *z* = 6.21), with more errors for nonword sequences (20.7%) than word sequences (11.4%).

#### “Different” trials

Restricting the analysis to trials requiring a “different” response, reference lexicality again affected error rates (*b* = 0.28, SE = 0.10, *z* = 2.69). In addition, type of change strongly influenced error rates (*b* = 0.82, SE = 0.11, *z* = 7.77) with almost two times more errors in the transposed (44.4%) than the replaced (23.4%) conditions. Critically, the reference lexicality × type of change interaction was also significant (*b* = 1.17, SE = 0.14, *z* = 8.50), with larger transposition effects (i.e., transposed minus replaced) for words (31.4%: *b* = 1.70, SE = 0.11, *z* = 16.2) relative to nonwords (10.4%: *b* = 0.52, SE = 0.09, *z* = 5.64).

### Discriminability (d’)

**Fig. 3:**
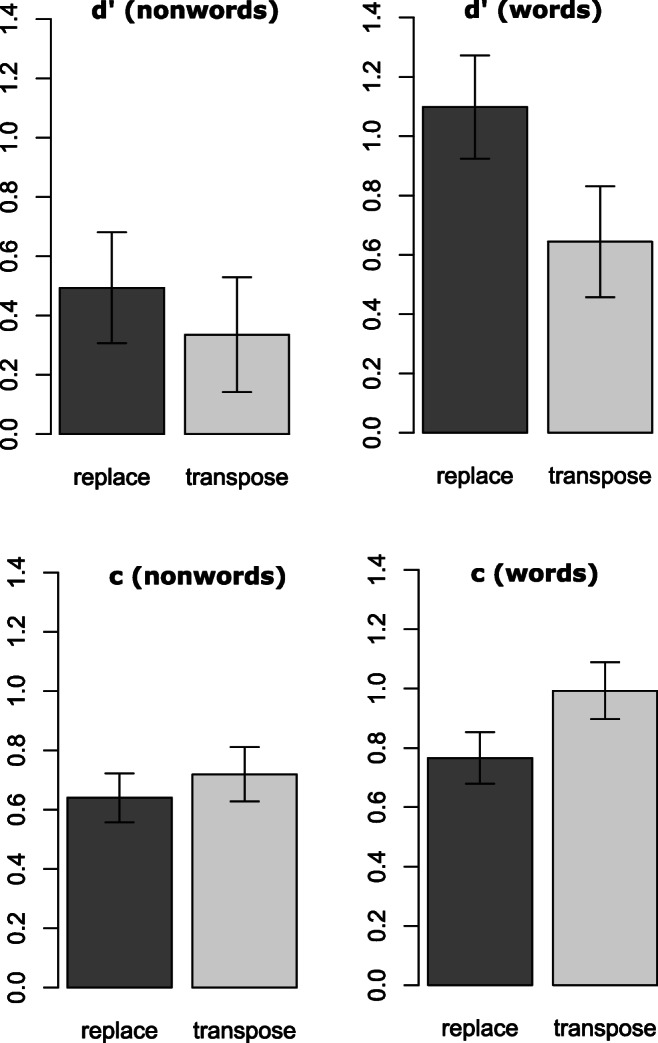
Signal detection theory discriminability (d’) and bias (*c*) values for nonwords (left) and words (right) for the two types of change (replace vs. transpose). Error bars represent 95% confidence intervals

In this analysis “hits” were defined as trials with a correct “different” response (i.e., a correct change detection), and false alarms were when a “different” response was incorrectly given on a “same” response trial (i.e., a false change detection). Sensitivity (d’) was calculated for each participant and for each condition (Type of change × Reference lexicality) as the z-score of hits minus the z-score of false alarms. The d’ values per participant, type of change, and reference lexicality were then entered as the dependent variable in a by-participant LME analysis. Condition means are shown in Fig. 3. Results revealed main effects of reference lexicality (*b* = 0.60, SE = 0.08, *z* = 7.95; d’ words = 0.87 vs. d’ nonwords = 0.41), a main effect of type of change (*b* = 0.15, SE = 0.07, *z* = 2.09; d’ transpose = 0.49 vs. d’ replace = 0.80), and a reference lexicality × type of change interaction (*b* = 0.30, SE = 0.11, *z* = 2.75). As can be seen in Fig. 3, the effect of type of change was greater for words (d’ replaced – d’ transposed = 0.45: *b* = 0.45, SE = 0.04, *z* = 10.9) than nonwords (d’ replaced – d’ transposed = 0.16: *b* = 0.16, SE = 0.03, *z* = 4.79).

### Bias (c)

In this analysis, *c* was defined as the distance between the “neutral point” (i.e., where signal and noise distributions cross) and the hypothetical response criterion, and was calculated as the average of the z-score of hits and the z-score of false alarms. Positive values reflect an overall tendency to answer **“**same**”** and negative values a tendency to respond **“**different**.”** We calculated *c* per participant for each type of change and reference lexicality. We then entered these values as the dependent variable in a by-participant LME analysis. Condition means are shown in Fig. 3. Results revealed a main effect of reference lexicality (*b* = 0.13, SE = 0.03, *z* = 3.60; *c* words = 0.88 vs. *c* nonwords = 0.68), main effect of type of change (*b* = 0.08, SE = 0.03, *z* = 2.27; *c* transpose = 0.86 vs. *c* replace = 0.70), and a reference lexicality × type of change interaction (*b* = 0.15, SE = 0.05, *z* = 2.99). As can be seen in Fig. 3, the effect of type of change on the bias measure was greater for words (*c* transposed – *c* replaced = 0.23: *b* = 0.23, SE = 0.02, *z* = 10.9) than nonwords (*c* transposed – *c* replaced = 0.08: *b* = 0.08, SE = 0.02, *z* = 4.79).

## Discussion

The present study applied the change-detection task, traditionally used to investigate visual short-term memory (e.g., Luck, 2008), in order to test the hypothesized spatiotopic nature of word-position encoding during reading. Our prior research had revealed a transposed-word effect in an immediate same-different matching paradigm, whereby it was harder for participants to decide that two briefly presented sequences of words were different when the difference was caused by changing the order of two adjacent words compared with a condition where the same two words were replaced with different words. Crucially, in this prior work we found transposed-word effects for ungrammatical sequences, hence pointing to a key role for bottom-up word identification processes in transposed-word effects, rather than uniquely top-down mechanisms driven by syntactic constraints. Given the hypothesized spatiotopic nature of word-position encoding, we expected to find the same pattern in the change-detection paradigm with concurrent articulation. We expected that the combination of the brief parallel presentation of to-be-remembered stimuli plus the 1-s delay before test and concurrent articulation during that period would provide an ideal means to investigate the spatiotopic nature of parallel word location encoding. We predicted that transposition effects should be greater with word sequences compared with nonwords sequences. The results obtained in our analyses of RT, accuracy, and d’ are in line with these predictions. Concerning the SDT analysis, we note that there were also significant main effects and an interaction in the analysis of response bias (*c*). Participants were more inclined to respond “same” when the reference was a word compared with nonword references, and this was particularly the case when the target differed from the reference by a transposition.^4^

One primary motivation for the present study was the finding reported by Mirault and Grainger (2020) that accurate grammatical decisions can be made to sequences of five words presented for only 300 ms and immediately followed by a backward mask. We interpreted this finding as reflecting parallel processing of word identities and their association with spatiotopic locations in VSTM. The rapid association of word identities to spatiotopic locations in VSTM would then enable the construction of a syntactic structure based on the parts-of-speech associated with each word (Deckerck, Wen, Snell, Meade, & Grainger, 2020), and this process of syntactic computation could continue after masking of the words. In our prior work, we interpreted transposed-word effects as reflecting parallel processing of word identities, and their noisy association with spatiotopic locations in VSTM (Mirault et al., 2018; Pegado & Grainger, 2019, 2020; Snell & Grainger, 2019b). We therefore expected to observe a transposed-word effect in the change-detection task. On the other hand, we reasoned that serial models of reading (e.g., Reichle et al., 1998; Reichle, Liversedge, Pollatsek, & Rayner, 2009) would have to appeal to the short-term storage of lower-level visual or orthographic information in order to account for the findings of Mirault and Grainger (2020). This account therefore predicted that there should be similar transposition effects for sequences of words and sequences of nonwords. Although we did find transposition effects with sequences of nonwords, these effects were much smaller than the effects seen with word sequences (see Figs. 2 and 3). Nevertheless, the fact that there was evidence for transposition effects with sequences of nonwords suggests that sublexical information such as letter identities or letter combinations can be stored in VSTM and used to perform the change-detection task. Even a limited amount of such information distributed across several nonwords would enable detection of a change, and the noisy nature of location encoding would generate the observed transposition effects.

In conclusion, the present results provide further evidence in support of parallel word processing and the spatiotopic nature of word-position encoding during reading (Snell et al., 2018). Our results also support the hypothesis that the noisy association of word identities with spatiotopic locations is one main source of transposed-word effects. The use of the change-detection task combined with concurrent articulation helped rule out other forms of short-term storage as the locus of transposed-word effects, and points to spatiotopic representations in VSTM as the mechanism that provides information about word positions that is essential for determining word order and the computation of syntactic structure for reading comprehension. Future research could fruitfully compare performance to the same set of stimuli in both change detection, as a standard VSTM task, and tasks traditionally used to investigate the storage of phonological representations in verbal STM. Such research would help clarify the distinction, hypothesized in the present work, between orthographic representations as the privileged means of storing verbal information in VSTM compared with phonological representations in vSTM.

## Footnotes

1 See Kennedy and Pynte (2008) for an earlier appraisal of the importance of spatial information for reading, and notably in order to keep track of word order when readers’ eye movements do not respect that order.

2 We used our previous study with the immediate same-different matching task (Pegado & Grainger, 2019) to ensure that we had sufficient power to reveal the critical interaction between type of change and reference lexicality in the present experiment. We calculated the power of that experiment using the SIMR package in R (Green & MacLeod, 2016). With 28 participants and the same number of items per condition as the present study, the estimated power was between 98.17% and 100% (95% confidence interval) to detect both the main effect of type of change and the type of change × lexicality interaction for accuracy on the “different” trials. We therefore judged that 31 participants would provide ample power in the present study to reveal the effects of interest.

3 Word frequencies were obtained from Lexique2 (New, Pallier, Brysbaert, & Ferrand, 2004) using the Zipf scale of van Heuven, Mandera, Keuleers, & Brysbaert (2014), which corresponds to Log10 number of occurrences per billion.

4 Note that this does not compromise the effects seen in discriminability since the calculation of d’ factors out any effect of bias.
